# Effect of Dietary Curcumin Supplementation on Duck Growth Performance, Antioxidant Capacity and Breast Meat Quality

**DOI:** 10.3390/foods10122981

**Published:** 2021-12-03

**Authors:** Sanjun Jin, Hao Yang, Fangju Liu, Qian Pang, Anshan Shan, Xingjun Feng

**Affiliations:** 1 Institute of Animal Nutrition, Northeast Agricultural University, Harbin 150030, China; Sanjunjin@163.com (S.J.); yanghao951209@163.com (H.Y.); liufangju0701@163.com (F.L.); pangqian1210@163.com (Q.P.); asshan@neau.edu.cn (A.S.); 2 Centre of Sport Nutrition and Health, Zhengzhou University, Zhengzhou 450001, China

**Keywords:** dietary curcumin, duck, growth performance, antioxidation, meat quality

## Abstract

This study aimed at examining the effects of curcumin supplementation on growth performance, antioxidant capacity, and meat quality of ducks. To investigate these effects, 600 healthy ducks were randomly assigned to four treatment groups with 10 replicates pens, and each pen contained 15 ducks. Ducks were fed a diet containing curcumin at levels of 0, 300, 400, and 500 mg kg^−1^ in different groups. The results demonstrated that curcumin supplementation is beneficial to the growth performance (*p* < 0.05) of ducks and antioxidant capacity (*p* < 0.05) of duck meat. In addition, dietary curcumin raised the meat quality of ducks, improving the meat color, increasing water-holding capacity, and inhibiting lipid and protein oxidation. In conclusion, the present study provides important insights into both the nutrient and qualities of ducks, finding that a dietary inclusion of 400–500 mg/kg of curcumin (kg^−1^) has the greatest effect.

## 1. Introduction

Duck meat is a popular product and is widely consumed throughout the world particularly in Asia [1]. In addition, duck meat is highly susceptible to oxidation because it has a high content of polyunsaturated fatty acids and essential amino acids. Lipid oxidation causes rancidity in meat [2] and has a positive relationship with protein oxidation [3]. Jin et al. (2020) reported that adding curcumin to the diet of ducks increased their antioxidant capacity, showing inhibited lipid and protein oxidation in their meat [4].

Curcumin, derived from turmeric rhizomes (*Curcuma Longa* Linn), was used as a coloring and flavoring agent and an antioxidation additive and is used in livestock as a feed additive [5,6,7]. Curcumin supplementation enhanced the meat quality of broilers by their increasing antioxidant capacity, maintaining cell membrane integrity, and increasing water-holding capacity [8]. Sahin et al. (2012) reported that curcumin supplementation activated the Nrf2/HO-1 pathway, upregulated antioxidation capacity, and increased the feed intake and growth performance of quail [9]. Similar studies reported that supplementing the diet with antioxidants improved growth performance and meat quality, and increased animals antioxidant capacity [10,11,12,13]. Khan et al. (2012) reported that curcumin protected the integrity of cell membranes against lipid and protein oxidation and decreased the cross-linking and particle size of proteins [14]. Daneshyar (2012) reported that supplementing the diet with turmeric (5 mg/kg^−1^) increased the meat quality of chicken by increasing their antioxidation capacity, suppressing lipid oxidation, and maintaining the contents of volatile compounds [15].

The aim of this study was to examine and evaluate the effects of curcumin supplementation on growth performance, antioxidant capacity, and meat quality. This study offers insights into the application of curcumin in livestock and poultry farming and the food industry.

## 2. Materials and Methods

### 2.1. Chemicals

Curcumin was obtained from Nanjing Nutri-herb Biotech Co., Ltd. (Nanjing, China, CAS: 458-37-7) with 99% purity.

### 2.2. Birds and Husbandry

One-day-old male ducks (*Anas platyrhynchos*, 600) were assigned to four groups: ducks fed with diet containing curcumin at different levels 0 (T_0_, control group), 300 (T_300_), 400 (T_400_), and 500 mg kg^−1^ (T_500_). Ducks were fed in cages (150 cm × 60 cm × 70 cm), and randomly assigned to 10 cages per treatment (15 ducks per cage). Their daily diet intake was monitored, and their weight was measured at regular intervals throughout the research.

### 2.3. Sample Collection

At 70 days of age, all ducks fasted for 12 h, ten ducks from each group were randomly selected and slaughtered. In order to evaluate the changes in meat quality, the entire pectoralis major muscle of each duck was taken, and then stored for 24 h at 4 °C.

### 2.4. Antioxidant Enzyme Assay

The duck meat was chipped and mixed in PBS (the mass ratio of sample to PBS was 1:9; 4 °C, 0.01 M, pH = 7.2–7.4). An analysis of the total superoxide dismutase (T-SOD), glutathione peroxidase (GPx), and catalase (CAT) activity in the liver was conducted using commercial diagnostic kits (Nanjing Jiancheng Bioengineering Institute, Nanjing, China).

### 2.5. Determination of pH and Color

The duck meat pH value was measured using a pH metre electrode (HI9125; Hanna Instruments, Padova, Italy). The duck meat color was determined using a colorimeter (Minolta CR-400; Konica Minolta, Tokyo, Japan). Ducks breast muscles were evaluated for changes in colour that three times in different locations of each sample.

### 2.6. Shear Force Assay

The shear forces (N) of samples were measured according to the process used by Jin et al. (2021) [16]. Two strips (1 ×1.27 × 10 cm) were cut parallel to the duck breast muscle fibres. Shear forces were evaluated using a texture analyser (Stable Micro System; TA: XT2i, Surrey, UKcity, England) with HDP/BSW (Warner-Bratzler Blade, Stable Micro System; TA: XT2i, Surrey, UK), which was attached to a load cell with a weight of 25 kg, the greatest force value of the sample was recorded.

### 2.7. Determination of Drip Loss

Drip loss was calculated following the process used by Jin et al. (2021) [16]. Duck samples were trimmed and weighed (W1, the original weight), then placed into a box for 24 h at 4 °C. The sample was removed, blotted dry, and weighed (W2, final weight) after 24 h at 4 °C. The drip loss was calculated as follows:

$$\mathrm{Drip}\;\mathrm{loss}\;\left(\%\right)=\frac{W_{1}-W_{2}}{W_{1}}\times 100$$

### 2.8. Determination of Cooking Loss

Cooking loss was calculated following the process used by Jin et al. (2021) [16]. The meat sample (2 × 5 × 4 cm) was carefully weighed, then placed into a thin-walled plastic bag and maintained in a water bath (85 °C); it was taken out when the internal temperature of the sample was 75 °C. The duck meat was blotted with filter paper and weighed again when the internal meat temperature cooled to 25 °C.

### 2.9. Determination of Water Mobility and Distribution

The water in samples was measured at room temperature (25 °C). The duck breast muscle sample (1 × 1 × 2 cm^3^) was placed in an 18 mm nuclear magnetic resonance tube and determined using the Minispecmq 20 ^1^H low-field nuclear magnetic resonance (LF-NMR) analyser (Bruker Optik GmbH, Ettlingen, Germany) at a static magnetic field strength of 0.47 T. One-dimensional (^1^H) spectra of the relaxation times (*T2b*, *T21*, and *T22*) were acquired using the Carr–Purcell–Meiboom–Gill (CPMG) pulse sequence. The raw data were normalized, analysed by the CONTIN algorithm, and represented by a continuous distribution of exponentials in a decay curve. 

### 2.10. Thiobarbituric Acid Reactive Substance (TBARS) Assay

The TBARS value was calculated following the process used by Han et al. (2019) [17] with small alterations. Duck breast muscles were individually chopped using a micro-waring blender for 30 s. The chopped sample (4.00 g) was placed into a 25 mL screw-cap test tube; and mixed with three drops of antioxidant solution (BHA), 3 mL of TBA solution, and 17 mL of TCA-HCl solution. The mixture was vortexed and then heated in a 100 °C boiling water bath for 30 min. After the reaction, the 5 mL solution (at room temperature) was mixed with 5 mL of chloroform for 1 min, and then centrifuged at 1800× *g* for 10 min. The supernatant was centrifuged again under the same conditions, and then the obtained absorbance of the supernatant was read at 532 nm. 

### 2.11. Determination of Carbonyl Content

The carbonyl sample was calculated following the process used by Mercier et al. (1988) [18]. The absorbance of the solution at 370 nm was measured, and the carbonyl content was evaluated according to the absorption coefficient of protein hydrazones $1.25\times 10^{6}$ L M^−1^ cm^−1^ according to the following formulas:

$$\mathrm{Carbonyl}\;\mathrm{content}\;\left({\mathrm{mmol}\;\mathrm{mg}}^{-1}\mathrm{prot}\right)=\frac{\left(A_{370}-A_{370\;\mathrm{Blank}}\right)}{22\times 0.5\times C}\times 1.25\times 10^{6}$$

where A_532_ is the solution measured at 532 nm, C is the sample protein concentration (mg prot L^−1^), and 1.25 × 10^6^ was the absorption coefficient of protein hydrazones.

### 2.12. Myofibrillar Protein (MP) Sulfhydryl Content Assay

The total sulfhydryl (SH) and reactive sulfhydryl contents in MP were determined by 5,5-dithiobis (2-nitrobenzoic acid) (DNTB) reagent according to the process used by Wang et al. (2020) [19]. The values of total sulfhydryl and reactive sulfhydryl were calculated as follows:

$$\mathrm{Total}\;\mathrm{sulfhydryl}\;\mathrm{content}\;\left(\mathrm{mmol}/g\right)=\frac{A_{412}\times 10^{6}}{1.36\times 10^{4}}$$

$$\mathrm{Reactive}\;\mathrm{sulfhydryl}\;\mathrm{content}\;\left(\mathrm{mmol}/g\right)=73.53\times \left(A_{412}-\;1.6934\times A_{540}+\;0.009923\right)$$

where A_412_ and A_540_ are the solution absorbances of the sample at 540 nm.

### 2.13. Determination of Myofibrillar Protein Solubility

The MP solution (1 mg mL^−1^, 5 mL) (15 M 1,4-piperazinediethane sulfonic acid (PIPES), 0.6 M NaCl, pH = 6.25) was centrifuged (10,000× *g*, 20 min, 4 °C). The supernatant of the MP solution was added into 4 mL of Biuret reagent and remained at 25 °C for 30 min. The MP solution dispersion solubility was expressed as:

$$\mathrm{MP}\;\mathrm{solubility}\left(\%\right)=\frac{S_{1}}{S_{2}}\times 100\%$$

where S_1_ is the MP solubility after centrifugation at 10,000× *g* for 20 min, and S_2_ is the original MP solubility in the MP solution.

### 2.14. Determination of Volatile Compounds

The volatile compounds of samples were extracted and detected according to the process used by Jin et al. (2021) [16]. Meat (3.00 g) was mixed with internal standard substance (IS) and placed into a headspace vial with PTFE/silicone septum. The extraction was performed at 45 °C for 40 min in a solid-phase microextraction (SPME) with a 75 µm carboxen/polydimethylsiloxane (CAR/PDMS) fibre, after sample equilibration 25 min at 45 °C. Volatile compounds extraction in a GC/MS (gas chromatography/mass spectrometry) system was conducted, then desorbed at 230 °C for 3 min. Then, desorption was set to 40 °C for 2 min, raised to 100 °C at 10 °C/min, and ultimately raised for 240 °C at 18 °C min^−1^ and held for 6 min under helium as carrier gas (1.0 mL min^−1^). The ion source temperatures were set to 230 °C. The mass spectrometer was scanned in the range of 33 to 450 *m*/*z*.

### 2.15. Statistical Analysis

The data were collected three times to obtain the mean value in each sample (ten ducks (samples) in each group), then analysed statistically using one-way ANOVA test in SPSS (Version 24.0, SPSS Inc., Chicago, IL, USA) using Duncan’s multiple comparison test at a 5% probability level. Statistical significance was indicated by *p* < 0.05. Curve estimation including linear and quadratic responses was made by assessing the orthogonal polynomial contrasts for effects of curcumin supplementation on growth performance and meat quality in *Anas platyrhynchos*.

## 3. Results

### 3.1. Growth Performance of Ducks

The effects of dietary curcumin on the growth performance of ducks are shown in Table 1. Dietary curcumin significantly increased the final weight, weight gain, and feed intake of ducks in curcumin groups relative to those in the T_0_ group (*p* < 0.05); however, there were no significant differences between the curcumin-supplemented groups (*p* > 0.05). In addition, there was no significant difference in F/C between the curcumin groups and the control group (*p* = 0.828). 

### 3.2. Antioxidant Enzyme

**Figure 1:**
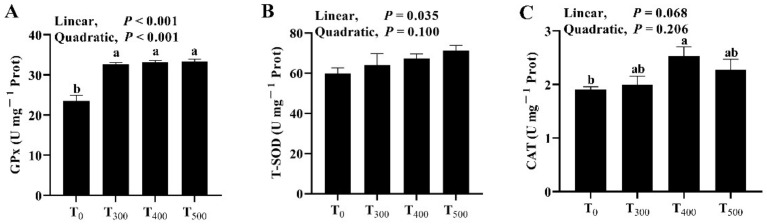
Effect of dietary curcumin on the antioxidation capacity of the duck breast muscle. (**A**) Effect of dietary curcumin on the GPx activity of the duck breast muscle. (**B**) Effect of dietary curcumin on the T-SOD activity of the duck breast muscle. (**C**) Effect of dietary curcumin on the CAT activity of the duck breast muscle. T_0_: ducks fed with the basal diet; T_300_, T_400_, and T_500_: ducks fed with 300, 400, and 500 mg of curcumin in kg^−1^ of basal diet. GPx, glutathione peroxidase; T-SOD, total superoxide dismutase; CAT, catalase. ^a, b^ Values with different letter above the column mean significant difference (*p* < 0.05).

As shown in Figure 1, although there was a significant increase in GPx activity (Figure 1A, *p* = 0.002) in the duck samples among groups, there was no significant difference in GPx activity in ducks breast muscle among the curcumin-supplemented groups (*p* > 0.05). Curcumin supplementation had no effect on T-SOD activity (Figure 1B, *p* = 0.288) and CAT activity (Figure 1C, *p* = 0.088); it also had no significant impact on the activities of T-SOD (*p* = 0.771) and CAT (*p* = 0.201) in the breast muscle of ducks among curcumin-supplemented groups.

### 3.3. Meat Quality

#### 3.3.1. Changes in Meat Colour

As shown in Table 2, dietary curcumin significantly increased *a* * values at 24 h (*p* = 0.002) and significantly decreased the *L* * value at 24 h (*p* = 0.002); however, there were no significant differences for the *a* * value (*p* = 0.775), *L* * value at 15 min (*p* = 0.147), nor *b* * values at 15 min (*p* = 0.254) and 24 h (*p* = 0.141) in the breast muscle of ducks.

#### 3.3.2. pH Values Changes

As shown in Table 2, the pH value at 15 min (*p* = 0.002) significantly increased with curcumin supplementation, but there were no significant differences in pH values at 24 h (*p* = 0.084). 

#### 3.3.3. Changes in Shear Force, Drip Loss, Cooking Loss and Water Distribution

**Figure 2:**
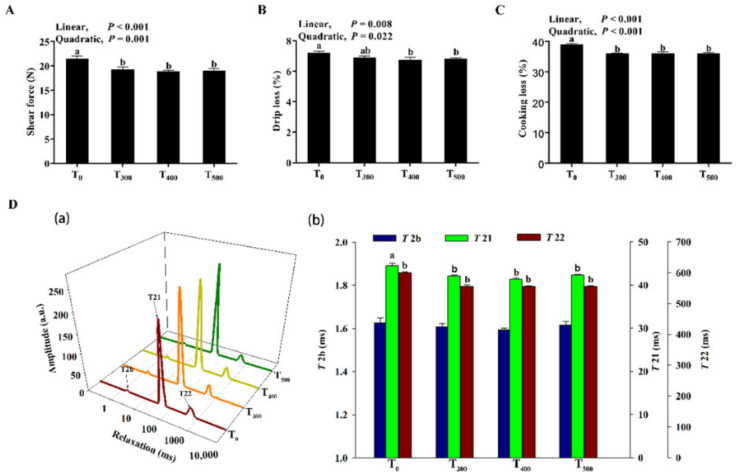
Effect of curcumin supplementation on duck breast muscle. (**A**) Shear force at 24 h. (**B**) Drip loss at 24 h. (**C**) Cooking loss at 24 h. (**D**) Influence of curcumin supplementation on *T2* relaxation times of duck breast muscle (**a**), and changes in the *T2* relaxation times (**b**); *T2b*: minor component between 0 and 10 ms; *T21*: major component between 10 and 100 ms; *T22*: third component between 100 and 1000 ms. ^a, b^ Values with different letter above the column mean significant difference (*p* < 0.05).

As shown in Figure 2A, curcumin supplementation significantly decreased shear force (*p* < 0.05). As shown in Figure 2B, C, drip loss (*p* = 0.049), and cooking losses (*p* = 0.006) significantly decreased with curcumin supplementation. As shown in Figure 2D, a decreasing *T2b* trend was observed in the samples with curcumin supplementation, but this was not a significant difference (*p* > 0.05). However, there were linear and quadratic decreases for *T21* and *T22* with curcumin supplementation (*p* < 0.01), and the *T21* and *T22* values were lowest in the T_500_ group.

### 3.4. Changes of TBARS, Carbonyl and Sulfhydryl Contents

**Figure 3:**
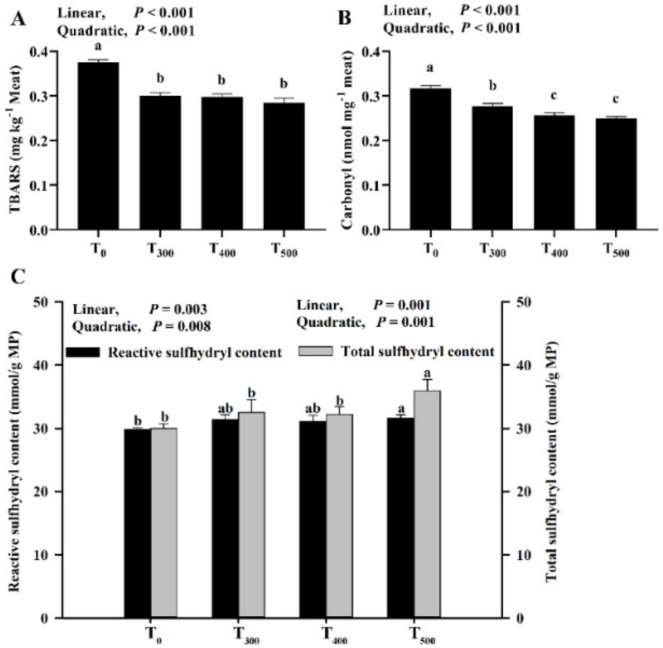
Effect of curcumin supplementation on lipid and protein oxidation and sulfhydryl content in duck breast muscle. (**A**) TBARS (thiobarbituric acid reactive substance). (**B**) Carbonyl (carbonyl was to evaluate the protein oxidation). (**C**) Reactive and total sulfhydryl was to evaluate the deformation of the protein. ^a, b, c^ Values with different letter above the column mean significant difference (*p* < 0.05).

As showcased in Figure 3A, there were significantly decreased TBARS values with curcumin supplementation (*p* < 0.05) after 24 h at 4 °C, but no significant difference in the TBARS value was observed among the curcumin-supplemented groups (*p* > 0.05). As depicted in Figure 3B, the carbonyl content in the sample decreased with a linear (*p* < 0.001) and quadratic (*p* < 0.001) response with curcumin supplementation, and a significant decrease in carbonyl content among curcumin-supplemented groups was observed (*p* < 0.05). As demonstrated in Figure 3C, the reactive and total sulfhydryl contents in MP significantly increased with curcumin supplementation and were the highest in the T_500_ group. 

### 3.5. Solubility of Myofibrillar Protein

**Figure 4:**
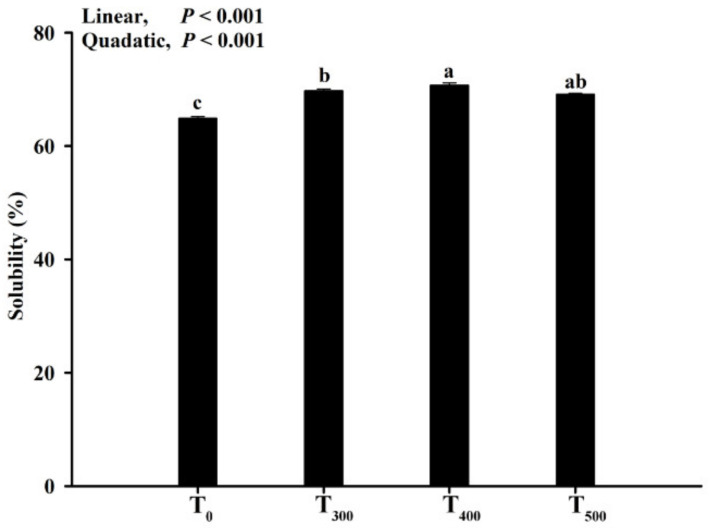
Effect of curcumin supplementation on the protein solubility of the duck breast muscle. Solubility represents proteins dissolved in the solvent. ^a, b, c^ Values with different letter above the column mean significant difference (*p* < 0.05).

As depicted in Figure 4, MP solubility increased (linear, *p* < 0.01; quadratic, *p* < 0. 01) with curcumin supplementation, but there was no significant difference among the curcumin-supplemented groups (*p* > 0.05). MP solubility was the highest in the T_400_ group.

### 3.6. Particle Size of Myofibrillar Protein

**Figure 5:**
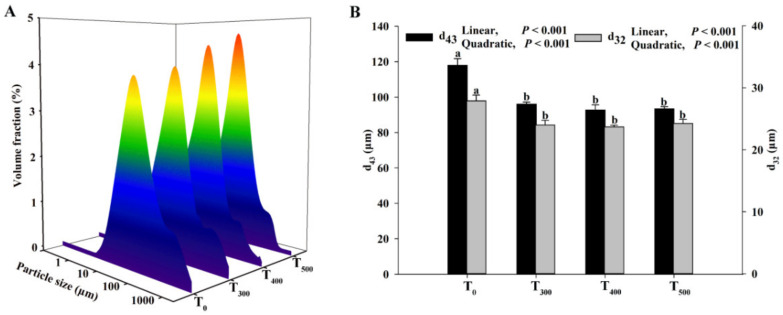
Effect of curcumin supplementation on the protein particle size of the duck breast muscle. (**A**) The average particle size (μm) was used to evaluate the size of protein. (**B**) d_43_ (volume-mean diameter), d_32_: volume-surface mean diameter. ^a, b^ Values with different letter above the column mean significant difference (*p* < 0.05).

As shown in Figure 5, the average particle diameter ranges of the myofibrillar protein were mainly concentrated in 1–1000 µm. The *d_43_* (volume-mean diameter) and *d_32_* (volume-surface mean diameter) decreased with curcumin supplementation, and a significant difference in the *d_43_* and *d_32_* of duck breast muscle in the curcumin-supplemented groups was observed (*p* < 0.05). The particle size of the myofibrillar protein was lowest in the T_500_ group.

### 3.7. Volatile Compounds Content

As shown in Table S2 (Supplementary Materials), 56 volatile compounds—within the samples—were extracted from the samples using SPME. The concentrations of aldehydes significantly decreased with curcumin supplementation (*p* < 0.05). Significant decreases in ketone concentrations occurred with curcumin supplementation (*p* < 0.05). The concentrations of most alcohol types (1-Pentanol, 1-Octen-3-ol,1-Hexanol, cyclostyle alcohol, 3-Octanol, 2-Decen-1-ol, 2-methyl-cyclohexanol, 4-(1,1-dimethylethyl), (E)-trans-2-Undecen-1-ol, and 2,3-Butanediol) were enriched with dietary curcumin levels (*p* < 0.05), except for three alcohols (2-ethyl, 1-Octanol, and 1-Hexanol) (*p* < 0.05). Curcumin supplementation linearly and quadratically inhibited the generation of acids (*p* < 0.05), except for dodecanoic acid and octanoic acid. The ester concentration was enhanced with curcumin supplementation (*p* < 0.05). Silicide concentrations decreased significantly with curcumin supplementation (*p* < 0.05). The volatile compounds including furan, alkanes, alanine, alkenes, and sulfide, and allyl methyl increased significantly with curcumin supplementation (*p* < 0.05).

## 4. Discussion

In this study, adding curcumin into the diet significantly increased WG and FI, and had an insignificant effect on the F/C of ducks with curcumin supplementation. In comparison with ducks in ochratoxin A (2 mg/kg^−1^), dietary curcumin increased the growth performance of ducks fed corn contaminated with ochratoxin A [20]. Abd El-Hack et al. (2021) reported that biological curcumin can improve the growth performance of broilers increasing nutrient intake and antioxidant capacity [21]. In addition, similar studies demonstrated that supplementing the diet with curcumin enhanced growth performance by increasing antioxidation capacity and improving the intestinal integrity of poultry, such as in Arbor Acres broiler chickens [22], Ross broiler chickens [12], wenchang broiler chickens [8], and quail [9].

Antioxidation enzymes such as SOD, GPx and CAT are important components of the antioxidation system. GPx, SOD and CAT are the first line of cell defence against free radicals and reactive oxygen species (ROS) and are indispensable in the defence strategy of antioxidants in the body [23]. The results reveal curcumin-supplemented diet have the ability to enhance the body’s antioxidation capacity in this study. This finding corroborates the results of another study that found dietary curcumin enhanced the antioxidant ability of laying hens by increasing SOD, T-AOC, CAT and GPx activities under heat-stressed environmental conditions [24]. Similarly, Jin et al. (2021) reported curcumin supplementation in the diet increased the activity of antioxidant enzymes of ducks fed Aflatoxin B1 [25].

The meat quality of livestock and poultry is mainly reflected by indicators such as colour, tenderness and water-holding capacity. Meat colour is an important indicator of consumer acceptance. Changes in meat colour may be related to the antioxidant and water-holding capacities. The increases in the *a* * value and decreases in *L* * and *b* * values for finishing pigs and ducks were accompanied by antioxidation capacity upregulation [16,26]. In this study, the improvement in duck meat quality alongside the increase in the a * value and the decrease in *L* * and *b* * values may be related to the upregulation of antioxidant capacity induced by curcumin supplementation. The increase in meat quality of transport-stress-impaired meat may be related to the increased antioxidant properties caused by resveratrol supplementation in the diet of the broilers [27].

The pH value is another indicator that is used to evaluate meat quality. In this study, curcumin supplementation significantly inhibited the reduction in pH values among curcumin groups compared to the control group; this phenomenon may be related to changes in the antioxidant capacity. Supplementing the diet with antioxidants increased pH values in the meat of pigs and ducks—as the antioxidation capacity of animals was upregulated [16,28]. Shear force is an indicator of meat tenderness, reflecting the content and nature of connective tissue and the chemical structural state of the myofibrillar protein in muscle. In this study, increasing the levels of dietary curcumin in ducks significantly reduced shear force values. Meng et al. (2020) reported that supplementing the diet with antioxidants decreased the shear force levels of pork as the antioxidation capacity increased and improved meat quality [28]. Drip loss is an indicator used to evaluate the sample water-holding capacity. Consumers are reluctant to accept meat with a high drip loss percentage. In our present study, dietary curcumin significantly decreased drip and cooking losses in duck breast muscle. Zhang et al. (2015) demonstrated that dietary curcumin decreased cooking and drip losses in broilers breast muscle, in line with our results [29]. In this study, decreases in drip and cooking losses occurred as antioxidant capacity increased in duck breast muscle. A similar study showed that supplementing the diet with curcumin significantly decreased the drip loss, and increased meat redness values of pigs [30]. In addition, water mobility and distribution in meat can be evaluated using low field-nuclear magnetic resonance (LF-NMR), which reflects the water state in a sample. The alteration of the water protein was associated with an increase in the mechanical damage and denaturation of proteins [31]. Cheng et al. (2016) reported increased drip loss—correlated with lipid peroxidation—destroyed the cell membrane [32]. A longer value means that the water is more fluidic [33]. Curcumin supplementation significantly decreased the *T21* and *T22* in duck breast muscle in this study. Zhang et al. (2019) reported that an increase in the TBARS value occurred with an increase in *T21* and *T22* relaxation in the porcine longissimus muscles (lumborum) which was in line with the results in this study [34].

MDA is a biomarker and secondary product of lipid oxidation that can be evaluated using TBARS values. Lipid oxidation of meat and meat products is a common event during the post-mortem period. Jin et al. (2021) reported that a significant decrease in the protein carbonylation of duck breast muscle occurred during the post-mortem period when the animal’s diet was supplemented with resveratrol [35]. Our previous study demonstrated that supplementing the diet with antioxidants inhibited lipid and protein oxidation [4,36]. Similarly, Karami et al. (2011) reported that 0.5% dietary turmeric powder-fed (5 g turmeric powder/kg dry matter intake added instead to the roughage part of a diet) goat decreased the MDA content in goat meat, which was vacuum-packed and refrigerated under retail conditions for 0, 7 or 14 days at 4 °C [37]. Protein carbonyl accumulation in meat demonstrated that muscle protein underwent oxidative reactions during the post-mortem period. Therefore, protein carbonyls produced in meat and meat products is an indicator to assess the levels of protein oxidation [30]. The results in this research were consistent with another study reporting that supplementation dietary curcumin decreased broiler chicken meat carbonyl contents after storage [22]. In beef steaks, protein oxidation led to the aggregation of myosin owing to bisulfide bonds cross-linking the protein [38], in line with the results in this study. Protein oxidation and aggregation were attributed to a decrease in protein solubility. Li et al. (2019) reported that MP showed more solubility with decreasing protein oxidation [39]. The results in this study were supported by changes in the protein carbonyls and sulfhydryl contents in duck meat.

MP particle size is an important indicator in the evaluation of protein denaturation. A previous study used *d*_32_ to describe the protein surface, which was related to the conformational changes in proteins caused by protein folding [40]. An increase in the protein particle size was observed to occur alongside the oxidation of meat during storage [38]. Another similar study revealed that mulberry polyphenols in dried minced pork slices weakened the protein cross-linking and aggregation, inhibiting protein denaturation during heat processing and storage [41].

In this study, most of the volatile compounds were identified in Beijing roasted ducks [42,43]. As presented in Table S2, the concentration of 56 volatile compounds in meat indicated that lipid oxidation was effectively inhibited by dietary curcumin. The changes in volatile compounds can also be proven by TBARS and carbonyl values. During storage, the number of alcohol types generated from lipid oxidation was higher in curcumin groups relative to those in the control group. However, the ester and acid concentrations were lower in that curcumin inhibited lipid and protein oxidation. Aldehydes were secondary products in the lipid oxidation process. Significant decreases in nonanal and hexanal (*p* < 0.01) occurred in the curcumin groups. Furan, 2-pentyl, has an important role in meat aroma and contributes sweet and buttery flavours to meat [44]. The furan content in the curcumin group was increased compared with that in the control group. Changes in volatile compounds in the duck breast may be related to dietary curcumin, in that supplementing the diet with antioxidants delayed the oxidation process and inhibited the generation of volatile compounds in tissues at an early post-mortem stage for animals owing to the higher antioxidant capacity [45]. Cantharidin, as a potent anticancer small molecule, is used in traditional Chinese medicine [46,47]. Interestingly, cantharidin was identified in duck meat for the first time and only found in ducks fed a basal diet supplementation with 500 mg of curcumin kg^−1^. However, the mechanism of the generation of cantharidin in duck meat of 500 mg of curcumin kg^−1^ of the basal diet group requires further investigation.

## 5. Conclusions

Curcumin supplementation enhanced duck’s growth performance and meat quality, protecting the protein against cross-linking bisulfide bonds and maintaining a lower MP particle size for a longer post-mortem period in duck breast muscle. Most volatile compounds decreased as curcumin supplementation increased. The results of the present study showed curcumin supplementation at a level of 400–500 mg/kg feed can improve duck meat quality by limiting the extent of lipid oxidation during the post-mortem period.

## Figures and Tables

**Table 1:** Effects of dietary curcumin on growth performance of ducks.

Items	Groups				SEM	*p*-Value	*p*-Value	
	T_0_	T_300_	T_400_	T_500_			Liner	Quadratic
IF, g	33.94	33.93	33.98	33.87	0.81	0.999	-	-
FW, g	1255.51 ^b^	1341.96 ^a^	1353.26 ^a^	1362.01 ^a^	33.72	0.018	0.002	0.006
WG, g	1221.57 ^b^	1308.04 ^a^	1319.28 ^a^	1328.14 ^a^	33.33	0.018	0.001	0.005
FI, g	4779.55 ^b^	5050.54 ^a^	5066.60 ^a^	5129.95 ^a^	80.52	0.020	0.001	0.001
F/C, *g*/*g*	3.92	3.87	3.84	3.86	0.087	0.828	0.385	0.657

IF, FW, WG, FI and F/C represent the means of 10 replicates. IF = initial weight; FW = final weight; FI = feed intake; WG = weight gain; F/CR = ratio of feed to weight gain. “-”means no significant difference among groups, and curve estimation including linear and quadratic responses wasn’t made by assessing the orthogonal polynomial contrasts. ^a, b^ Values with different letter superscripts within the same row mean significant difference (*p* < 0.05). SEM = standard error (SE) of means.

**Table 2:** Effects of dietary curcumin on color and pH of duck breast muscle.

Items	T_0_	T_300_	T_400_	T_500_	SEM	*p*-Value	*p*-Value	
							Linear	Quadratic
Colour parameters								
L *_15 min_	39.60	38.25	38.16	37.30	0.544	0.147	0.020	0.070
L *_24 h_	45.60 ^a^	44.00 ^ab^	42.34 ^b^	43.23 ^b^	0.006	0.002	0.002	0.006
a *_15 min_	15.31	17.49	18.14	18.72	0.880	0.775	<0.001	<0.001
a *_24 h_	14.56 ^b^	15.68 ^b^	15.80 ^b^	15.98 ^a^	0.334	0.002	<0.001	0.001
b *_15 min_	5.20	5.11	4.91	4.88	0.183	0.254	0.010	0.191
b *_24 h_	5.18	4.90	4.84	3.76	0.155	0.141	0.027	0.063
pH								
pH_15 min_	5.71 ^b^	5.81 ^ab^	5.92 ^a^	5.94 ^a^	0.076	0.019	0.002	0.008
pH_24 h_	5.13	5.30	5.31	5.33	0.081	0.084	0.013	0.034

Values of “*L* *” “*a* *” and “*b* *” represent brightness, redness and yellowness of meat surface. ^a, b^ Values with different letter superscripts within the same row mean significant difference (*p* < 0.05).

## Data Availability

The data used and/or analysed in this study are available from the corresponding author on reasonable request.

## Supplementary Materials

The following are available online at https://www.mdpi.com/article/10.3390/foods10122981/s1, Table S1: Ingredient composition and nutrient content of the basal diet (%, as-fed basis), Table S2: Concentration (ng g^−1^) of volatile compounds identified and quantified by gas chromatography/mass spectrometry in the duck breast muscle with different dietary curcumin.
