# Supplementary material for: Effect of Dietary Curcumin Supplementation on Duck Growth Performance, Antioxidant Capacity and Breast Meat Quality
Source: Foods. 2021 Dec 3;10(12):2981. doi: 10.3390/foods10122981 (PMC8701154; doi:10.3390/foods10122981)
Supplement: Supplementary file 1 [file foods-10-02981-s001.zip › foods-1448064-supplementary.pdf]

Table S1. Ingredient composition and nutrient content of the basal diet (%, as-fed basis).

**Items**	**1-4 Weeks**	**5-8 Weeks**	**9-10 Weeks**
Ingredient			
Corn (7.9)	61.70	68.94	75.80
Soybean meal (45)	26.09	26.80	20.10
Corn protein flour (55)	7.90	—	—
Dicalcium phosphate	1.40	1.40	1.40
Limestone	1.08	1.06	1.06
Salt	0.38	0.38	0.38
DL-Methionine	0.15	0.22	0.16
L-Lysine	0.20	0.10	0.00
choline chloride (50%)	0.10	0.10	0.10
Premix	1.00[1]	1.00[2]	1.00[3]
Total	100	100	100
Nutritional level			
Calculated nutrient [4]			
Metabolizable energy (MJ/kg)	12.14	11.98	12.21
CP (%)	20.67	17.51	15.03
Calcium (%)	0.90	0.90	0.88
Total phosphorus (%)	0.68	0.67	0.65
Non-phytate phosphorus (%)	0.44	0.44	0.44
Lysine (%)	1.07	0.95	0.71
Methionine (%)	0.48	0.48	0.39
Methionine +cystine (%)	0.81	0.75	0.63
Threonine (%)	0.75	0.66	0.56
Tryptophane (%)	0.21	0.19	0.16

[1] The premix provided per kilogram diet: vitamin A 4000 IU, vitamin D3 2000 IU, vitamin E 20 mg, vitamin K3 2.0 mg, vitamin B1 2.0 mg, vitamin B2 12 mg, vitamin B6 3.0 mg, vitamin B12 0.02 mg, nicotinic acid 50 mg, D-pantothenic acid 10 mg, folic acid 1 mg, biotin 0.2 mg, Cu 8 mg, Fe 60 mg, Mn 100 mg, Zn 60 mg, Se 0.2 mg, I 0.4 mg.

[2] The premix provided per kilogram diet: vitamin A 3000 IU, vitamin D3 2000 IU, vitamin E 10 mg, vitamin K3 2.0 mg, vitamin B1 1.5 mg, vitamin B2 8 mg, nicotinic acid 30 mg, D-pantothenic acid 10 mg, vitamin B6 3.0 mg, vitamin B12 0.02 mg, biotin 0.1 mg, folic acid 1 mg, Cu 8 mg, Fe 60 mg, Mn 80 mg, Zn 40 mg, Se 0.2 mg, I 0.4 mg.

[3] The premix provided per kilogram diet: vitamin A 2500 IU, vitamin D3 1000 IU, vitamin E 10 mg, vitamin K3 2.0 mg, vitamin B1 1.5 mg, vitamin B2 8 mg, nicotinic acid 30 mg, D-pantothenic acid 10 mg, vitamin B6 3.0 mg, vitamin B12 0.02 mg, biotin 0.1 mg, folic acid 1 mg, Cu 8 mg, Fe 60 mg, Mn 80 mg, Zn 40 mg, Se 0.2 mg, I 0.3 mg.

[4] Values were calculated based on the data provided by Feed Database in China (2004).

Table S2. Concentration (ng g^{-1}) of volatile compounds identified and quantified by gas chromatography/mass spectrometry in the duck breast muscle with different dietary curcumin.

Volatile Compounds (ng g^{-1})	Groups				SEM	*p-Value*	*p-Value*		CAS
	T_0	T_{300}	T_{400}	T_{500}			Linear	Quadratic	
Aldehydes									
Nonanal	22.15 ^{a}	6.93 b	6.83 ^{b}	6.51^b	0.194	< 0.001	0.027	< 0.001	124-19-6
Hexanal	20.49 ^{a}	15.49 ^{b}	10.65 ^{c}	3.16 ^{d}	0.275	< 0.001	0.000	< 0.001	66-25-1
Benzaldehyde	2.93 ^{a}	0.00 ^{b}	0.00 ^{b}	0.00 ^{b}	0.045	< 0.001	0.033	< 0.001	100-52-7
Hexadecanal	2.82 ^{a}	0.00 ^{b}	0.00 ^{b}	0.00 ^{b}	0.043	< 0.001	0.033	< 0.001	629-80-1
Tetradecanal	4.21 ^{a}	0.00 ^{b}	0.00 ^{b}	0.00 ^{b}	0.037	< 0.001	0.033	< 0.001	124-25-4
Pentadecanal	4.70 ^{a}	0.00 ^{b}	0.00 ^{b}	0.00 ^{b}	0.010	< 0.001	0.033	< 0.001	2765-11-9
2-Octenal, (E)-	6.66 ^{a}	0.00 ^{b}	0.00 ^{b}	0.00 ^{b}	0.061	< 0.001	0.033	< 0.001	2548-87-0
2-Decenal, (E)-	3.33 ^{a}	0.00 ^{b}	0.00 ^{b}	0.00 ^{b}	0.201	< 0.001	0.035	< 0.001	3913-81-3
2,4-Nonadienal, (E,E)-	2.90 ^{a}	0.00 ^{b}	0.00 ^{b}	0.00 ^{b}	0.041	< 0.001	0.033	< 0.001	5910-87-2
2,4-Decadienal, (E,E)-	28.62 ^{a}	0.00 ^{b}	0.00 ^{b}	0.00 ^{b}	0.618	< 0.001	0.033	< 0.001	25152-84-5
2-Nonenal, (E)-	5.10 ^{a}	0.00 ^{b}	0.00 ^{b}	0.00 ^{b}	0.082	< 0.001	0.033	< 0.001	18829-56-6
Benzaldehyde, 3-hydroxy-4-methoxy-	3.40 ^{a}	0.00 ^{b}	0.00 ^{b}	0.00 ^{b}	0.082	< 0.001	0.033	< 0.001	621-59-0

Ketones									
2,3-Octanedione	60.07 [a]	24.49 [b]	20.33 [c]	17.02 [d]	1.297	< 0.001	0.008	< 0.001	585-25-1
Acetoin	3.82 [a]	3.56 [b]	3.50 [b]	2.83 [c]	0.057	< 0.001	< 0.001	< 0.001	513-86-0
Alcohols									
1-Pentanol	3.61 [d]	4.91 [c]	4.15 [b]	7.80 [a]	0.100	< 0.001	0.006	0.002	71-41-0
1-Hexanol	3.30 [a]	3.17 [b]	3.71 [c]	7.32 [d]	0.111	< 0.001	< 0.001	< 0.001	111-27-3
1-Octen-3-ol	42.20 [d]	43.93 [c]	44.93 [b]	46.03 [a]	0.142	< 0.001	< 0.001	< 0.001	1832-68-4
1-Octanol	5.75 [a]	2.44 [b]	2.37 [c]	2.67 [c]	0.076	< 0.001	< 0.001	< 0.001	111-87-5
Cyclooctyl alcohol	0.00 [b]	0.00 [b]	0.00 [b]	2.82 [a]	0.043	< 0.001	0.033	< 0.001	696-71-9
3-Octanol, 2-methyl-	2.71 [d]	3.55 [c]	8.46 [b]	12.54 [a]	0.217	< 0.001	< 0.001	< 0.001	26533-34-6
1-Hexanol, 2-ethyl-	4.74 [a]	2.72 [b]	2.61 [b]	2.24 [c]	0.089	< 0.001	< 0.001	< 0.001	104-76-7
Cyclohexanol, 4-(1,1-dimethylethyl)-	5.60 [c]	5.72 [c]	11.91 [a]	11.32 [b]	0.241	0.002	0.002	0.003	98-52-2
2-Decen-1-ol, (E)-	2.49 [d]	5.52 [c]	5.80 [b]	7.81 [a]	0.081	< 0.001	< 0.001	< 0.001	18409-18-2
trans-2-Undecen-1-ol	2.87 [c]	2.94 [b]	3.82 [b]	5.52 [a]	0.084	< 0.001	< 0.001	< 0.001	75039-84-8
2,3-Butanediol	3.23 [d]	3.47 [c]	6.01 [b]	7.00 [a]	0.069	< 0.001	< 0.001	< 0.001	513-85-9
Acids									
Butanoic acid	4.61 [a]	2.33 [b]	1.92 [c]	1.61 [d]	0.087	< 0.001	0.004	< 0.001	107-92-6

Hexanoic acid	6.95 [a]	6.01 [b]	5.72 [c]	5.69 [c]	0.096	0.002	0.007	< 0.001	142-62-1
Octanoic acid	3.2	3.24	3.13	3.23	0.083	< 0.001	0.857	0.746	124-07-2
Nonanoic acid	3.23 [a]	2.48 [b]	2.40 [c]	2.18 [d]	0.026	< 0.001	0.001	0.000	112-05-0
n-Decanoic acid	5.14 [b]	5.55 [a]	4.56 [c]	4.58 [c]	0.019	< 0.001	0.016	0.055	334-48-5
Benzoic acid	6.71 [a]	4.12 [b]	3.59 [c]	2.53 [d]	0.055	< 0.001	< 0.001	0.000	65-85-0
Dodecanoic acid	3.89	3.83	3.78	3.77	0.062	0.276	0.044	0.146	143-07-7
n-Hexadecanoic acid	21.37 [a]	17.89 [b]	15.17 [c]	14.02 [d]	0.081	< 0.001	< 0.001	< 0.001	57-11-4
Tetradecanoic acid	2.17 [a]	0.00 [b]	0.00 [b]	0.00 [b]	0.009	< 0.001	< 0.001	< 0.001	544-63-8
Acetic acid	6.58 [a]	6.57 [a]	6.27 [b]	5.20 [c]	0.088	< 0.001	< 0.001	< 0.001	64-19-7
Esters									
Hexanoic acid, methyl ester	10.89 [a]	7.48 [b]	7.52 [b]	7.13 [b]	0.339	< 0.001	< 0.001	< 0.001	6624-60-8
Nonanoic acid, methyl ester	0.00 [c]	3.22 [b]	4.72 [a]	4.80 [a]	0.084	< 0.001	< 0.001	< 0.001	1731-84-6
Octanoic acid, methyl ester	6.33 [c]	7.80 [b]	7.99 [ab]	8.09 [a]	0.092	< 0.001	0.010	< 0.001	10152-76-8
iso-Amyl levulinate	8.41 [d]	9.02 [c]	10.62 [b]	17.71 [a]	0.124	< 0.001	0.005	< 0.001	71172-75-3
9-Octadecenoic acid, methyl ester, (E)-	3.63 [b]	5.30 [a]	5.14 [a]	5.18 [a]	0.095	< 0.001	0.003	0.009	22147-34-8
1,2-Benzenedicarboxylic acid, bis(2-methylpropyl) ester	3.01 [c]	3.29 [b]	3.38 [b]	4.91 [a]	0.077	< 0.001	0.005	0.000	84-69-5
9-Hexadecenoic acid, methyl ester, (Z)-	2.71 [c]	3.19 [a]	2.80 [b]	3.21 [a]	0.038	< 0.001	0.026	0.089	1120-25-8

9,12-Octadecadienoic acid (Z,Z)-, methyl ester	3.52 [d]	5.44 [b]	4.69 [c]	5.64 [a]	0.059	< 0.001	< 0.001	0.001	112-63-0
Hexadecanoic acid, methyl ester	4.52 [b]	4.55 [b]	4.56 [b]	5.10 [a]	0.045	< 0.001	0.023	< 0.001	112-39-0
Alkenes								< 0.001	
2-Octen-1-ol, (E)-	6.03 [d]	10.39 [c]	11.42 [b]	14.13 [a]	0.13	< 0.001	< 0.001	< 0.001	18409-17-1
Anethole	0.00 [c]	0.00 [c]	2.38 [b]	5.83 [a]	0.036	< 0.001	0.002	< 0.001	4180-23-8
Hexadecane	0.00 [d]	6.44 [c]	8.20 [b]	9.41 [a]	0.043	< 0.001	< 0.001	< 0.001	629-73-2
Alkanes								< 0.001	
Tetradecane	2.76 [d]	3.67 [c]	3.80 [b]	4.53 [a]	0.054	< 0.001	< 0.001	< 0.001	629-59-4
Octadecane	2.06 [d]	3.53 [c]	4.11 [b]	10.96 [a]	0.052	< 0.001	0.003	< 0.001	593-45-3
Cantharidin								< 0.001	
Cantharidin	0.00 [b]	0.00 [b]	0.00 [b]	3.80 [a]	0.041	< 0.001	0.033	< 0.001	56-25-7
Silicide								< 0.001	
Cycloheptasiloxane, tetradecamethyl-	43.14 [a]	11.47 [b]	10.79 [c]	6.01 [d]	0.257	< 0.001	< 0.001	< 0.001	107-50-6
Cyclooctasiloxane, hexadecamethyl-	8.11 [d]	6.39 [c]	5.73 [b]	5.34 [a]	0.096	< 0.001	< 0.001	< 0.001	556-68-3
Cyclononasiloxane, octadecamethyl-	25.30 [a]	16.52 [b]	13.77 [c]	0.00 [d]	0.367	< 0.001	< 0.001	< 0.001	556-71-8
Alanine								< 0.001	
Alanine	0.00 [b]	0.00 [b]	0.00 [b]	7.32 [a]	0.123	< 0.001	0.033	< 0.001	56-41-7

Furan								< 0.001	
Furan, 2-pentyl-	2.44 [d]	3.60 [c]	3.90 [b]	4.27 [a]	0.089	< 0.001	< 0.001	< 0.001	3777-69-3
Sulfide								< 0.001	
Sulfide, allyl methyl	9.90	9.94	9.95	9.95	0.317	0.404	0.078	0.212	10152-76-8

SEM = Standard error of the means. Orthogonal polynomials were used to investigate linear and quadratic responses to the level of curcumin treatment. Results were means with n = 10 per group.
